# Surgical repair of distal arch psendoaneurysm from ruptured penetrating aortic ulcer with the frozen elephant trunk technique

**DOI:** 10.1186/1749-8090-9-68

**Published:** 2014-04-05

**Authors:** John Kokotsakis, Dimitrios Tassopoulos, Jacob Ttofi, Leanne Harling, Hutan Ashrafian, Konstantinos Velissarios, Theodore Kratimenos, Stratos Anagnostou, Thanos Athanasiou

**Affiliations:** 1 Department of Surgery and Cancer, Imperial College London, 10th Floor QEQM Building, St Mary’s Hospital Praed Street, London W2 1NY, UK; 2 Department of Cardiac Surgery Evangelismos Hospital, Athens, Greece

**Keywords:** Pseudoaneurysm, Aortic arch, Stented graft, Frozen elephant trunk

## Abstract

Ruptured Penetrating Ulcer and aortic arch pseudo-aneurysm is a rare condition but one which carries a high risk of rupture. We report the case of a 74-year-old man with aortic arch pseudo-aneurysm, in which a Frozen Elephant Trunk procedure was successfully performed. There were no postoperative complications at 6 months follow-up. The Computed Tomography Angiogram demonstrated thrombus formation in the pseudo-aneurysm lumen, with no endoleak on the stented part of the descending thoracic aorta and complete patency of all branches of aortic arch. This case demonstrates that the Frozen Elephant Trunk technique may be the treatment of choice when treating such complex aortic arch lesions provided there is no absolute contraindication to radical surgical intervention. However, long-term clinical efficacy and safety have yet to be confirmed.

## Background

Ruptured penetrating aortic ulcer (PAU) with psendoaneurysm formation is considered an acute aortic syndrome, a dissection variant, which requires emergency attention. PAU’s are predominantly situated in the descending aorta and less frequently in the arch, abdominal or ascending aorta [1]. Endovascular treatment of PAU’s located in the descending aorta has emerged as an attractive and successful option over the past decade [2,3]. However, for PAU’s located in the arch the type of intervention required is less well defined.

We report the surgical treatment of a patient with a ruptured PAU in the aortic arch using the frozen elephant trunk (FET) technique.

## Case presentation

A 74-year-old man with hypertension and COPD was emergently admitted with back pain and syncope due to a large 6.2 cm pseudo-aneurysm of the aortic arch originating from a PAU.

**Figure 1:**
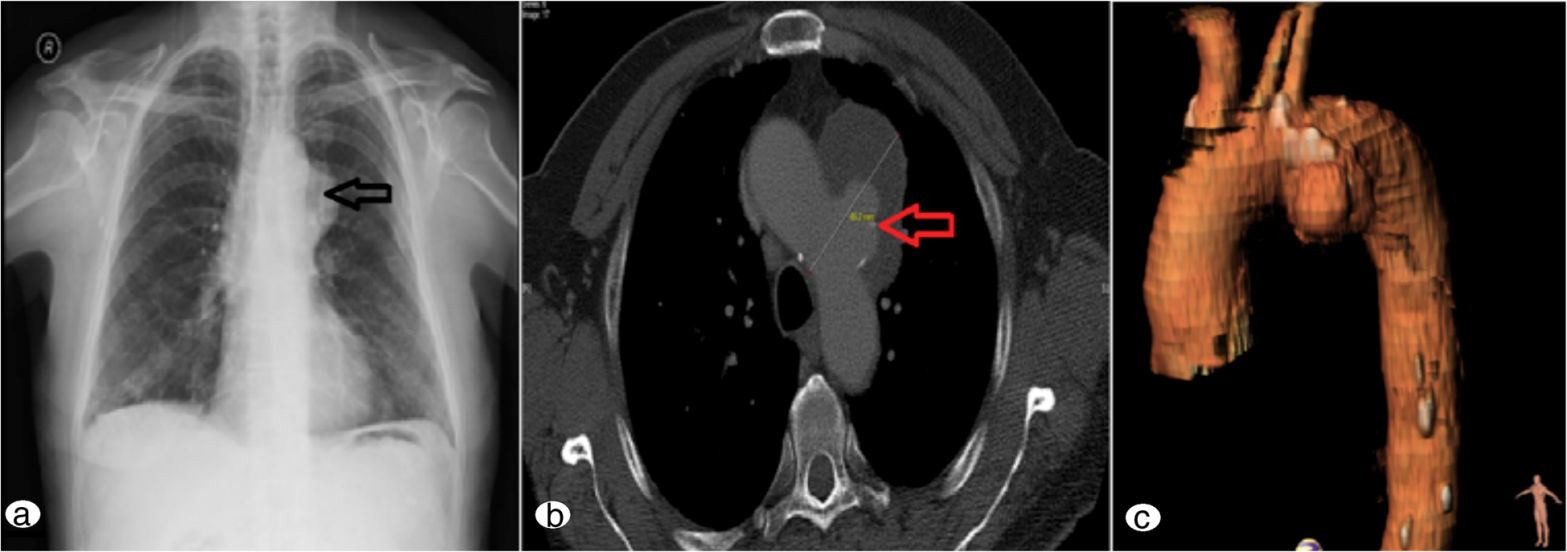
**Pre-operative imaging demonstrating the pseudo-aneurysm. (a)** Chest x-ray demonstrating dilatation of the aortic arch (black arrow); **(b)** CT angiogram (axial view) demonstrating the aortic arch pseudo-aneurysm; **(c)** CT angiogram showing the pseudo-aneurysm located in the medial part of the aortic arch.

Preoperative evaluation included a coronary angiogram that revealed normal coronary arteries. Transthoracic echocardiographic analysis showed mild (1+/4) aortic regurgitation with a left ventricular ejection fraction of 60%. Carotid triplex ultrasound scan showed no carotid artery stenosis. Chest radiograph and Computed Tomographic angiography (CTA) (Figure 1a, b and c) showed a 6.2 cm pseudo-aneurysm of the aortic arch involving the origin of the left subclavian artery (LSA), originating from a penetrating aortic ulcer (PAU) just opposite the ostium of the LSA in the lesser curvature of the arch. The ascending, descending and abdominal aorta were of normal size. Blood tests were unremarkable.

### Surgical technique

Anesthetic induction was achieved by our standard technique including administration of sodium pentothal, sevofluorane, fentanyl and muscle relaxant. Invasive monitoring included the use of right and left radial arterial lines, a pulmonary artery catheter and a Foley catheter with temperature probe to measure bladder temperature as an indicator of core body temperature. Cerebral monitoring was achieved by means of transcutaneous cerebral oximetry (INVOS 3100-SD; Troy, Mich) and electroencephalogram. A catheter for cerebrospinal fluid (CSF) drainage was inserted. Transesophageal echocardiography (TEE) was performed intraoperatively.

After systemic heparinization and before incision, a guide-wire was inserted through the right femoral artery in the descending thoracic aorta under fluoroscopic and TEE control.

A median sternotomy with left supraclavicular extension was performed. A right infraclavicular incision was also made and the right axillary artery (RAA) exposed. Cardiopulmonary bypass (CPB) was instituted with an arterial cannula introduced into the RAA through an interposed 8 mm Dacron graft and a single two-stage venous cannula introduced into the right atrium. The arterial line of CPB circuit was bifurcated, one arm for axillary artery perfusion and the other arm for later perfusion of the side branch of the arch graft. CPB was commenced and flow was maintained between 2.2 and 2.4 L.min^-1^. A retrograde cardioplegia catheter was placed in the coronary sinus via the right atrium. Left ventricular decompression was achieved with a vent catheter placed through the right superior pulmonary vein. Active cooling was started to a bladder temperature of 26°C. During cooling, the ascending aorta, proximal aortic arch and innominate artery were dissected and exposed without dividing the innominate vein. The LCCA and the LSA with the origin of the left vertebral artery were exposed in the left neck and left supraclavicular area avoiding contact with the fragile arch psendoaneurysm. Upon cardiac fibrillation, a cross clamp was placed across the ascending aorta and resected above the coronary ostia in the sinotubular junction. Myocardial arrest was achieved with cold crystalloid cardioplegia at 25 ml.kg^-1^ (Custodiol, Koehler CHEMIE, Alsbach-Haenlein, Germany) delivered both retrograde and antegrade through the coronary ostia.

Once the target bladder temperature of 26°C was reached, CPB flow was reduced to 1 l.min^-1^ and the innominate artery (IA), the LCCA and LSA were clamped in order to obtain selective unilateral antegrade cerebral perfusion (SACP). There was no need for additional perfusion of the LCCA and LSA due to adequate collateral circulation. With the rest of the body in circulatory arrest, the ascending aorta and proximal arch were resected close to the proximal margin of the LCCA origin. The IA was transected 1 cm distal from its origin, while the LCCA and LSA were transected in the left neck and left supraclavicular area just before the origin of left vertebral artery correspondingly. The proximal stumps of LCCA and LSA were ligated.

The soft guide-wire, previously inserted through the right femoral artery in the descending thoracic aorta was exchanged to a stiff guide-wire using a pigtail catheter. The hybrid stent-graft system (33 × 160 mm, E-vita open plus, Jotec Inc., Hechingen, Germany) was then introduced antegradely through the open arch in the descending aorta over the stiff guide-wire and released with a pull-back system. The proximal landmark for stent-graft placement was the origin of LCCA. After deployment the incorporated Dacron graft was pulled back, cut to a minimum and sutured to the transected arch with 3–0 polypropylene interrupted horizontal mattress sutures with external Teflon strip reinforcement. A Hegar dilator was inserted inside the E-vita prosthesis to check the opening of the stent-graft.

**Figure 2:**
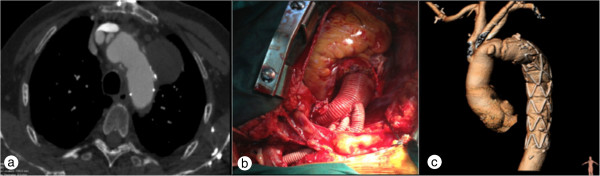
**Intra-operative photographs of surgical repair and post-operative imaging. (a)** CT angiogram axial view demonstrating postoperatively that the pseudo-aneurysm has been thrombosed; **(b)** Intra-operative photograph demonstrating total aortic arch replacement and anastomosis with head and neck vessels; **(c)** 3D-reconstucted CT angiogram demonstrating the frozen elephant trunk and patency of anastomoses of head and neck vessels.

A Dacron vascular prosthesis with four branches 28 × 10 × 8 × 8 × 10 mm (Tetrabranch Jotec) was then prepared and anastomosed with the cuff composed by the native aorta and the E-vita prosthesis. Antegrade systemic perfusion was restored through the 4^th^ perfusion branch (10 mm) of the graft. The 3^rd^ branch (8 mm) of the Dacron prosthesis was passed below the innominate vein and anastomosed to the LSA in an end-to-end fashion, and released to the circulation. The main trunk of the arch prosthesis was anastomosed to the ascending aorta, at the STJ level, terminating cardiac ischemia. Finally, the 2^nd^ (8 mm) and 1^st^ (10 mm) branches of the arch prosthesis were connected to the LCCA and IA, and after de-airing systemic circulation to the brain was restored, while SACP from the right axillary artery was stopped (Figure 2b).

After complete rewarming and de-airing of the heart CPB was terminated with minimal inotropic support. Atrial and ventricular pacing wires were placed. Protamine was administered and hemostasis achieved. The synthetic graft was covered with pericardium, excluding it from the sternal wound. Two chest tubes were inserted in the anterior mediastinum and the wounds closed in the standard fashion. The CPB time was 190 min, SACP time 115 min, lower body circulatory arrest time 40 min, myocardial ischemic time 95 min. Deployment of the stented part of the hybrid prosthesis required 10 min. The correct opening of the stent-graft was controlled with TEE.

The patient had an uneventful recovery and was extubated 8 hours after the operation. He remained in the intensive care unit for 2 days. A CTA scan was performed at 6 months after surgery (Figure 2a and c).

## Discussion

There is accumulating experience in the use of the frozen elephant trunk technique for the management of extended aortic aneurysms, however its role in the setting of aortic dissection and particularly pseudo-aneurysms of the arch and proximal descending thoracic aorta remains unclear [4-6]. Furthermore, variations in the anatomical location, urgency of presentation, prosthesis type, and extent of follow up have lead to difficulties in interpretation of the current evidence base. As such, the main learning point of our case is to demonstrate that a radical surgical approach such as the frozen elephant trunk technique may provide excellent outcomes in complex aortic pathology such as aortic arch pseudoaneurysm.

Pseudo-aneurysm of the aortic arch complicating an atheromatous penetrating aortic ulcer is a lethal and rare condition, which carries a high risk of rupture. The aortic tissue close to a pseudo-aneurysm of the medial aortic arch is usually very poor quality and the best treatment is to excise it. This makes other approaches including total arch replacement or conventional elephant trunk less optimal options for the following reasons: (1) The distal aortic anastomosis can be technically challenging due to mismatch between the graft and the proximal descending aorta; (2) Haemostasis and the risk of dissection makes stitching very challenging as the aortic wall is usually very thin and friable; (3) There is a high risk of thrombo-embolic complications as the large sac of the pseudo-aneurysm may contain a significant amount of atheroma, debris and clot.

One of the potential advantages of the frozen elephant trunk is that the use of the stented graft in the descending aorta expedites thrombus formation, whereas residual perfusion in the space around the graft in the conventional elephant trunk may lead to delayed thrombus formation and progressive aneurysmal dilatation [6]. Indeed, in their multi-centre report of patients treated with the FET technique, Tsagakis and colleagues demonstrate 97% of patients with acute dissection and 89% of those with chronic dissection had full thrombosis of their false lumen during the follow-up period [7]. Secondly, it has been emphasized that the flapping action of the downstream portion of the conventional elephant trunk may be responsible for peripheral embolism [8], a phenomenon that may be eliminated with the FET approach.

Surgical treatment of complex aortic arch pathology such as in this case commonly requires debranching of the innominate artery, left common carotid artery (LCCA) and left subclavian artery (LSA) in order to achieve an adequate landing and sealing zone for the graft. Whilst fenestrated or branched stent grafts are available, these are both expensive and custom made and thus cannot be used in the emergency setting. Alternative therapeutic options include total endovascular debranching of the aortic arch, or hybrid procedures combining surgical de-branching of the head and neck vessels with endovascular stenting of the aortic arch. Embolization of the pseudoaneurysm may also be perfomed with detachable coils or endovascular injection of embolic agents, however these were not options in our case because of the caliber of the pseudoaneurysm tear and the size of the pseudoaneurysm lumen.

There is evidence to suggest that endovascular repair is a better choice for higher risk, elderly or frail patients with significant comorbidities or where pathology is located in the descending thoracic aorta [9-11]. Hybrid procedures are associated with lower mortality and morbidity, however these techniques may be particularly technically challenging and may increase the risk of post-operative stroke and neurocognitive complications. As such, it is paramount to consider not only the reported outcomes of these different approaches but also patient selection, availability of equipment and surgical experience when selecting the most appropriate treatment strategy.

## Conclusions

In summary, this case demonstrates that the Frozen Elephant Trunk technique may be the treatment of choice and confer excellent mid-term outcomes when treating such complex aortic arch lesions provided there is no absolute contraindication to radical surgical intervention. However, the long-term clinical efficacy and safety have yet to be confirmed and should be addressed in future prospective clinical studies.

## Consent

Written informed consent was obtained from the patient for publication of this case report and any accompanying images. A copy of the written consent is available for review by the Editor-in-Chief of this journal.

## Abbreviations

PAU: Penetrating aortic ulcer; FET: Frozen elephant trunk; COPD: Chronic obstructive pulmonary disease; CTA: Computerised tomography angiography; LSA: Left subclavian artery; CSF: Cerebrospinal fluid; TEE: Trans esophageal echo; RAA: Right axillary artery; CPB: Cardiopulmonary bypass; LCCA: Left common carotid artery; IA: Innominate artery; SACP: Selective unilateral antegrade cerebral perfusion; STJ: Sino-tubular junction.

## Competing interests

There are no financial or non-financial competing interests.

## Authors’ contributions

JK, DT, TA and TK carried out the surgical procedure, with perfusion support provided by SA. KV provided the medical imaging. JK, TA, KV, TV, TK and SA participated in provision of clinical information and reviewed the manuscript. JT, LH and HA performed the literature review and drafted the manuscript. TA finalised the manuscript and discussion. All authors read and approved the final manuscript.
